# Resolvable Group State Estimation with Maneuver Based on Labeled RFS and Graph Theory †

**DOI:** 10.3390/s19061307

**Published:** 2019-03-15

**Authors:** Weifeng Liu, Yudong Chi

**Affiliations:** School of Automation, Hangzhou Dianzi University, Xiasha Higher Education Zone, Hangzhou 310018, China; cyd0418@163.com

**Keywords:** resolvable group target tracking, labeled random finite sets, GLMB, adjacency matrix

## Abstract

In this paper, multiple resolvable group target tracking was considered in the frame of random finite sets. In particular, a group target model was introduced by combining graph theory with the labeled random finite sets (RFS). This accounted for dependence between group members. Simulations were presented to verify the proposed algorithm.

## 1. Introduction

Multi-target tracking is widely used in defense and civilian fields. When multiple targets move in the air, they usually perform tasks in formation. The formation can be seen as group targets on a radar screen. When a shoal of fish swim and be detected by sonar. The shoal of fish usually exhibits the characteristics of group targets. In tracking space debris, it also shows the similar characteristics of group targets. Group target tracking can be seen as a special type of multi-target tracking problem. Most of the traditional target tracking algorithms are based on data association methods. When the number of targets increases, the computation time increases sharply. This has become an obstacle to the development of algorithms [1]. The Random Finite Set (RFS) method provides a new direction for research in multi-target tracking. It can avoid the process of data association and has become a research hotspot in the field of multi-target tracking [2,3]. The RFS method on the basis of the Bayes optimality [4,5] and multi-target estimation error [6] presents the theory of global target tracking in complex observation scenes through target set distribution. RFS method is already one of the important research directions of multi-target tracking [7] today. It can be deployed in a wide range of applications through a series of algorithms, such as the Probability Hypothesis Density (PHD) filter [8,9,10], Cardinalized PHD filter (CPHD) [11,12], multi-Bernoulli filter (MeMBer) [6], Generalized Labeled Multi-Bernoulli (GLMB) filter [13,14,15], and its multi-scan version [16,17]. In addition, Reference [18] represents a new breakthrough by demonstrating that the GLMB filter can track in excess of one million targets simultaneously, over one billion data points. It proposes an algorithm that can track more than one million targets per scan simultaneously. Different from these results, we considered the resolvable group tracking issue. This paper is an extended version of our conference paper (Reference [19]).

The PHD filter belongs to the moment approximation filter algorithm. It takes the first order statistical moment of the posterior probability of the multi-target state set to obtain a feasible approximate form. Further, a Gaussian Mixture PHD (GM-PHD) filter for linear Gaussian was proposed in Reference [10]. Subsequently, many scholars have studied the convergence problem [20,21], track consistency problem [22,23], and state extraction problem [24] of the PHD filters and made a series of breakthroughs. The CPHD filter [11] is a kind of high-order generalization of the PHD filter. The filter can simultaneously propagate the multi-target posterior PHD and posterior cardinality distribution, so the instantaneous estimation result of the number of targets is more stable and accurate than the PHD filter. When the PHD filter and CPHD filter are implemented by the seqence Monte Carlo (SMC) method, the state extraction process is complicated. Mahler proposed the MeMBer filter to solve this problem, which makes use of the existence probability and probability density of the target to make numerical approximation to the multi-target probability distribution function. It simplifies the process of state extraction. However, although the MeMBer filter simplifies the state extraction process, it overestimates the cardinality. Vo et al. proposed the Cardinality Balanced MeMBer (CBMeMBer) filter [25] to alleviate this problem. Then, Vo et al. proposed the GLMB filter [13,14] by introducing the label into the MeMBer filter. The GLMB filter inherits the advantages of MeMBer filter’s simple particle implementation and state estimation. At the same time, the GLMB filter does not need analytical approximation, which is different from the standard PHD, CPHD, and MeMBer filter. Standard PHD, CPHD, MeMBer filters use approximation techniques to ensure their conjugate distributions, which affects their estimation accuracy. Moreover, the GLMB filter can obtain target tracks through the label introduced. So, the GLMB filter can obtain the number of targets and their tracks at the same time, which is a very significant breakthrough. It has been proven to be a Bayes optimal filter [26]. Vo et al. also proposed an implementation method called the δ-GLMB filter, also known as the Vo-Vo filter. But the GLMB filter’s computational complexity increased sharply with the number of targets. In response to the problem, scholars proposed some improved algorithms, such as the Labeled Multi-Bernoulli (LMB) filter [27] and the Marginalized δ-GLMB (Mδ-GLMB) filter [28]. These approximations, however, still have the same numerical complexity as the GLMB filter. In their paper [15], Vo et al. combined the prediction and update steps into a single step and proposed an efficient implementation of the GLMB filter using Gibbs sampling. Here, we used Gibbs GLMB to name it.

Because of these breakthroughs, the RFS approach has been applied in many fields. In addition to information fusion and target tracking already mentioned, RFS method also has deep application in other fields. For example, in Reference [29], the RFS method was applied to machine learning; in References [30,31,32,33,34], the RFS method was applied to computer vision. In References [35,36], the RFS method was applied to an autonomous vehicle. In addition, RFS technology has also been applied to sensor scheduling [37,38,39,40,41,42,43,44], sensor networks [45,46], track-before-detect, tracking of merged measurements, extended targets, and group targets [47,48,49,50,51].

In this paper, multiple targets with similar motion states and certain cooperative relationships are called the group target. The group target is very similar to the extended target in some aspects, such as dynamic modeling, state estimation, and potential estimation. This similarity stems from the fact that both the group target and the extended target will generate multiple measurements, and the multiple measurements have a certain spatial pattern. More importantly, in the multiple measurements generated by the group target and the extended target, the distance between the measurement points is less than the threshold of the tracking gate. This brings additional challenges to the application of traditional multi-target tracking algorithms.

When the measurements of the group target are located in the different resolution units of the sensor, the group target is called the resolvable group target. Conversely, if more than one measurement is located in the same resolution unit, it is called an unresolvable group target. In this paper, we focused on the resolvable group. At present, the research results on the group target can be divided into two categories: the algorithm based on data association and the RFS. References [19,51,52] worked on the group target tracking filter based on the GLMB filter. Reference [51] considered the structure of the groups, but did not consider the impact of the cooperative relationship between group targets on the estimation, while References [19,52] made some work on collaborative noise.

## 2. Backgrounds

### 2.1. Labeled Random Finite Set (RFS)

Mahler introduced the theory of RFS to target tracking in a series of works [8,53,54,55], where the multi-target state at time *k* was represented by a finite-set:

(1) $$X_{k}=\left\{x_{k,1},\cdots ,x_{k,N_{k}}\right\}.$$

The uncertainty in a multi-target state is described by random finite set models that captures birth, spawning, death, and motion. The multi-target state transition equation is given by:

(2) $$X_{k}=\left[\underset{x\in X_{k-1}}{⋃}S_{k\left|k-1\right.}\left(x\right)\right]⋃\left[\underset{x\in X_{k-1}}{⋃}B_{k\left|k-1\right.}\left(x\right)\right]⋃\Gamma_{k},$$

where $S_{k\left|k-1\right.}\left(x\right)$ denotes the surviving targets, $B_{k\left|k-1\right.}\left(x\right)$ denotes the spawned targets, and $\Gamma_{k}$ denotes the birth targets. The multi-target measurement is the finite subset:

(3) $$Z_{k}=\left\{z_{k,1},\cdots ,z_{k,M_{k}}\right\}.$$

The theory of labeled RFS is given in References [13,14]. A labeled RFS is formed by augmenting a mark to the state of each target. In other words, we attach distinct labels $\ell \in L=\{\alpha_{i}:i\in N\}$ to different targets, where N is the set of positive integers. Labeled RFS requires that the labels of any two targets are different, i.e., the function:

(4) $$\Delta \left(X\right)=\left\{\begin{array}{c} 1,\left|L\left(X\right)\right|=\left|X\right|\\ 0,\left|L\left(X\right)\right|\neq \left|X\right| \end{array}\right.$$

must equal 1. The densities of an LMB RFS and a labeled Poisson RFS are given in Reference [13]. For the LMB RFS, its density is described as:

(5) $$\begin{array}{c} \pi \left(\left\{\left(x_{1},l_{1}\right),\cdots ,\left(x_{n},l_{n}\right)\right\}\right)=\delta_{n}\left(\left|\left\{l_{1},\cdots ,l_{n}\right\}\right|\right)\times \prod_{\zeta \in \Psi }\left(1-r^{\zeta }\right)\prod_{j=1}^{n}\frac{1_{\alpha (\Psi )}\left(l_{j}\right)r^{\left(\alpha^{-1}\left(l_{j}\right)\right)}\left(x_{j}\right)}{1+r^{\left(\alpha^{-1}\left(l_{j}\right)\right)}} \end{array}.$$

### 2.2. Graph Theory

A graph *G* consists of two sets, the set of vertices *V* and the set of edges *E* [56]. At time *k*, the graph can be described by $G_{k}=\left(V_{k},E_{k}\right)$ [52], where $V_{k},E_{k}$ a are non-empty finite set. If the edges have direction, the graph is a directed graph; conversely, it is an undirected graph.

**Figure 1:**
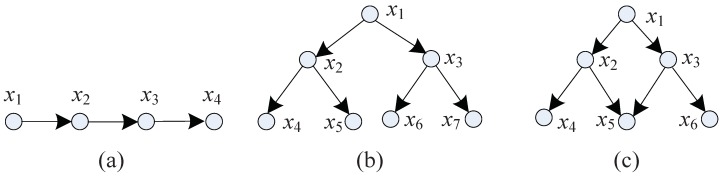
Resovable Group with various structures. (**a**) A chain structure; (**b**) tree structure; and (**c**) complex structure.

A group structure is similar to a graph structure, so we use the asymmetric adjacency matrix to describe the structure of the resolvable group target. This matrix can describe the collaborative relationship between the members of a resolvable group target, such as the parent-child relationship between mutually dependent targets. In the target adjacency matrix:

(6) $$A_{d}=\left[\begin{array}{cccc} 0 & a(1,2) & \cdots & a(1,n)\\ a(2,1) & 0 & \cdots & a(2,n)\\ \vdots & \vdots & \ddots & \vdots \\ a(n,1) & a(n,2) & \cdots & 0 \end{array}\right],$$

a(i,j)=1 means target *i* is the parent node for target *j*, and a(i,j)=0 means target *i* is target *j*’s child node, or target *i* has no relationship with target *j*. For example, the group structures in Figure 1 are described by the following asymmetric adjacency matrices:

(7) $$A_{d}^{a}=\left[\begin{array}{cccc} 0 & 0 & 0 & 0\\ 1 & 0 & 0 & 0\\ 0 & 1 & 0 & 0\\ 0 & 0 & 1 & 0 \end{array}\right],A_{d}^{b}=\left[\begin{array}{ccccccc} 0 & 0 & 0 & 0 & 0 & 0 & 0\\ 1 & 0 & 0 & 0 & 0 & 0 & 0\\ 1 & 0 & 0 & 0 & 0 & 0 & 0\\ 0 & 1 & 0 & 0 & 0 & 0 & 0\\ 0 & 1 & 0 & 0 & 0 & 0 & 0\\ 0 & 0 & 1 & 0 & 0 & 0 & 0\\ 0 & 0 & 1 & 0 & 0 & 0 & 0 \end{array}\right]A_{d}^{c}=\left[\begin{array}{cccccc} 0 & 0 & 0 & 0 & 0 & 0\\ 1 & 0 & 0 & 0 & 0 & 0\\ 1 & 0 & 0 & 0 & 0 & 0\\ 0 & 1 & 0 & 0 & 0 & 0\\ 0 & 1 & 1 & 0 & 0 & 0\\ 0 & 0 & 1 & 0 & 0 & 0 \end{array}\right].$$

## 3. Revolvable Group Tracking with Maneuver

### 3.1. Graph Theory Model of Labeled RFS

Let any vertex $v_{i}$ in the graph be a labeled state. For a group target, the set of vertices is finite, and we can define edges from the vertices set as follows:

(8) $$e_{i,j}:\left(x_{i},x_{j}\right)\to \left\{1,0\right\}.$$

Equation (8) indicates the edges are defined on labeled states. When the edges only depend on the labels, the definition reduces to:

(9) $$e_{i,j}:\left(l_{i},l_{j}\right)\to \left\{1,0\right\}.$$

Equation (9) shows that the graph only depends on the target labels. Hence, the structure of the group is encapsulated by the graph defined on the target labels.

### 3.2. Dynamic Model of Multiple Resolvable Group

**Figure 2:**
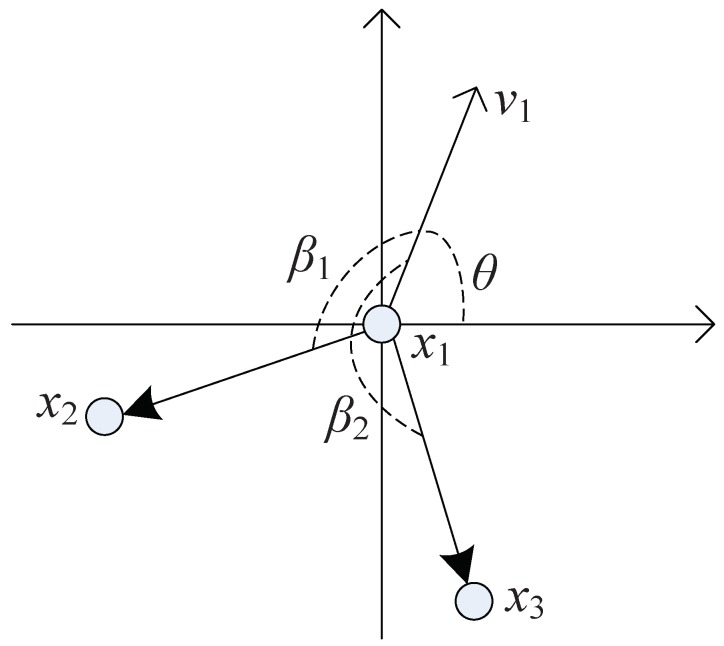
Angle of resolvable group target structure. The node $x_{1}$ is a root and also parent node of nodes $x_{2}$ and $x_{3}$. Target $x_{1}$ moves with velocity vector $v_{1}$, where the angle is θ against *x*-axis. Besides, the angle of the child node $x_{2}$ and its parent $x_{1}$ is $\beta_{1}$. Similarly, the angle between nodes $x_{1}$ and $x_{3}$ is $\beta_{2}$.

If the target has a single parent node, the resolvable group targets dynamic model [7] is given as follows:

(10) $$\begin{array}{ccc} x_{k+1,i} & = & F_{k,l}x_{k,l}+b_{k}\left(l,i\right)+\Gamma_{k,i}\omega_{k,i} \end{array}$$

(11) $$\begin{array}{ccc} z_{k+1,i} & = & H_{k+1}x_{k+1,i}+v_{k+1,i}, \end{array}$$

where $F_{k,l}$ denotes the state transition matrix, $\Gamma_{k,i}$ is the state noise factor matrix, $H_{k+1}$ is the observation matrix, $\omega_{k,i}$ is the process noise, and $\upsilon_{k+1,i}$ is observation noise. All these are assumed to be Gaussian. For the state $x_{k,i}=\left[p_{k,x}\left(i\right),{\dot{p}}_{k,x}\left(i\right),p_{k,y}\left(i\right),{\dot{p}}_{k,y}\left(i\right)\right]\in X_{k}$, $p_{k,x}\left(i\right)$ and ${\dot{p}}_{k,x}\left(i\right)$ are the position and velocity of target *i* on the *x*-axis, $p_{k,y}\left(i\right)$ and ${\dot{p}}_{k,y}\left(i\right)$ indicate the position and velocity of target *i* on the *y*-axis. For $b_{k}\left(l,i\right)$, *l* is the parent node for target *i*. It contains the direction and distance information between the parent and child nodes. For a resolvable group with fixed formation and structure, see Figure 2.

**Figure 3:**
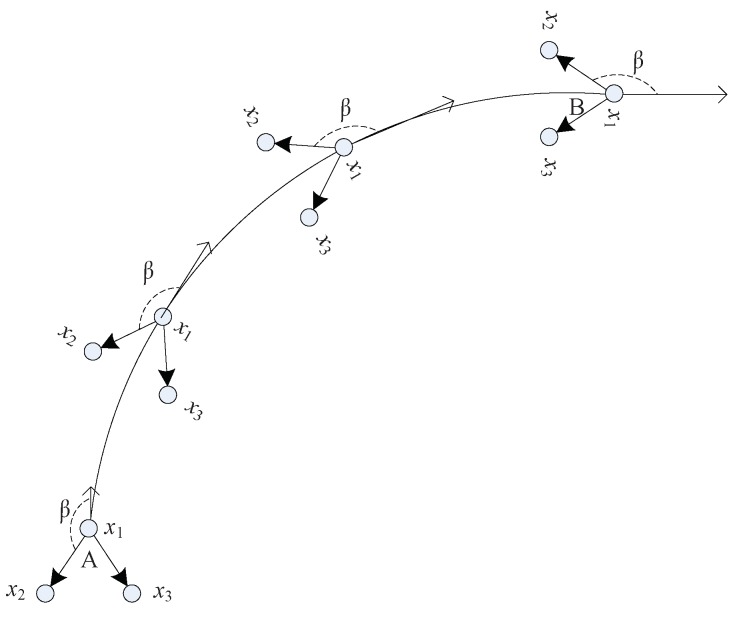
A formation moves in a constant turning (CT) from points *A* to *B*. To keep a fixed formation, the angle between two vectors: the vector for the velocity of parent node $x_{1}$ and the vector of positions between $x_{1}$ and $x_{2}$ should keep a fixed value β.

That is, the angle β(s,d) between the velocity vector $v_{s}$ of the group targets and the position vector $v_{d}$ between the parent and child nodes remain constant against time. This can be illustrated by Figure 3. Let the formation be three nodes: $x_{1},x_{2}$ and $x_{3}$, where $x_{1}$ is parent node and $x_{2}$ and $x_{3}$ are two child nodes. If the formation moves from Point *A* to *B* and keeps a fixed shape, two conditions should be met. First, the three nodes are with the same velocites. Second, the angle between two vectors, i.e., velocity vector of parent node $x_{1}$ and position vector between parent node and child nodes, should be remain unchanged. For instance, the angle β shown in Figure 3. Thefore, we assumed the angle β(s,d) is constant for a fixed formation.

At time *k*, the motion direction for the parent target is given by the angle:

(12) $$\theta_{k}\left(l,i\right)=\arctan\frac{\left[\begin{array}{cccc} 0 & 0 & 0 & 1 \end{array}\right]{x_{k,l}}^{T}}{\left[\begin{array}{cccc} 0 & 1 & 0 & 0 \end{array}\right]{x_{k,l}}^{T}}.$$

Therefore, for group targets, the displacement vector $b_{k}\left(l,i\right)$ can be represented as:

(13) $$\begin{array}{c} b_{k}\left(l,i\right)={\left[R_{k}\left(l,i\right)\cos\left(\theta_{k}\left(l,i\right)-\beta_{k}\left(l,i\right)\right),0,R_{k}\left(l,i\right)\sin\left(\theta_{k}\left(l,i\right)-\beta_{k}\left(l,i\right)\right),0\right]}^{T} \end{array},$$

where $R_{k}\left(l,i\right)$ denotes the designed distance between parent node *l* and child node *i*. $\beta_{k}\left(l,i\right)$ is the designed angle between nodes *l* and *i*. If the parent node $x_{k,l}$ moves in constant velocity (CV) mode and the group is with a fixed formation, then the distance variable $R_{k}\left(l,i\right)$ and angles $\theta_{k}\left(l,i\right),\beta_{k}\left(l,i\right)$ are all constant, i.e., $R_{k}\left(l,i\right)=R\left(l,i\right),\theta_{k}\left(l,i\right)=\theta \left(l,i\right),\beta_{k}\left(l,i\right)=\beta \left(l,i\right)$. Thus, the displacement vector will be a constant, as in:

(14) $$b_{k}\left(l,i\right)={\left[R_{k}\left(l,i\right)\cos\left(\theta_{k}\left(l,i\right)-\beta_{k}\left(l,i\right)\right),0,R_{k}\left(l,i\right)\sin\left(\theta_{k}\left(l,i\right)-\beta_{k}\left(l,i\right)\right),0\right]}^{T}.$$

Nevertheless, under a maneuvering motion model, the dynamic equation Equation (10) is nonlinear due to the displacment vector between node *i* and its parent node *l* dependent on the parent state $x_{k,l}$. Assume that the group has a fixed formation, so the vector can be written according to Equation (14):

(15) $$\begin{array}{c} b_{k}\left(l,i\right)={\left[R\left(l,i\right)\cos\left(\theta_{k}\left(l,i\right)-\beta (l,i)\right),0,R\left(l,i\right)\sin\left(\theta_{k}\left(l,i\right)-\beta (l,i)\right),0\right]}^{T}\\ =[R\left(l,i\right)\left(a_{\beta ,2}\left(l,i\right)\cos\theta_{k}\left(l,i\right)+a_{\beta ,1}\left(l,i\right)\sin\theta_{k}\left(l,i\right)\right),0,R\left(l,i\right){\left(a_{\beta ,2}\left(l,i\right)\sin\left(\theta_{k}\left(l,i\right)-a_{\beta ,1}\left(l,i\right)\right)\cos\left(\theta_{k}\left(l,i\right)\right),0\right]}^{T}, \end{array}$$

where $\sin(\theta_{k}\left(l,i\right)$ and $\cos(\theta_{k}\left(l,i\right)$ are given by:

(16) $$\begin{array}{c} \sin\left(\theta_{k}\left(l,i\right)\right)=\frac{{\dot{p}}_{k,y}\left(l,i\right)}{\sqrt{{\dot{p}}_{k,x}^{2}\left(l\right)+{\dot{p}}_{k,y}^{2}\left(l\right)}} \end{array}$$

(17) $$\begin{array}{c} \cos\left(\theta_{k}\left(l,i\right)\right)=\frac{{\dot{p}}_{k,x}\left(l\right)}{\sqrt{{\dot{p}}_{k,x}^{2}\left(l\right)+{\dot{p}}_{k,y}^{2}\left(l\right)}} \end{array}$$

(18) $$\begin{array}{c} a_{\beta ,1}\left(l,i\right)=\sin\left(\beta \left(l,i\right)\right) \end{array}$$

(19) $$\begin{array}{c} a_{\beta ,2}\left(l,i\right)=\cos\left(\beta \left(l,i\right)\right) \end{array}$$

Further, we can transfer Equation (15) to

(20) $$\begin{array}{c} b_{k}\left(l,i\right)=c_{k}\left(l,i\right)C_{a}\left(l,i\right)x_{k,l} \end{array}$$

(21) $$\begin{array}{c} c_{k}\left(l,i\right)=\frac{R(l,i)}{\sqrt{{\dot{p}}_{k,x}^{2}\left(l\right)+{\dot{p}}_{k,y}^{2}\left(l\right)}} \end{array}$$

(22) $$\begin{array}{c} C_{a}\left(l,i\right)=\left[\begin{array}{cccc} 0 & a_{\beta ,2}\left(l,i\right) & 0 & a_{\beta ,1}\left(l,i\right)\\ 0 & 0 & 0 & 0\\ 0 & a_{\beta ,1}\left(l,i\right) & 0 & -a_{\beta ,2}\left(l,i\right)\\ 0 & 0 & 0 & 0 \end{array}\right]. \end{array}$$

According to Equation (20), if $c_{k}\left(l,i\right)$ is a costant coefficient and $C_{a}$ is a constant matrix, then $b_{k}\left(l,i\right)$ can be seen as a linear transformation of the state $x_{k,l}$ dependent on some constants. In general, the constants are known in advance. For example, for a group with fixed formation and moving in a CT mode, the variables R(l,i), $\sqrt{{\dot{p}}_{k,x}^{2}\left(l\right)+{\dot{p}}_{k,y}^{2}\left(l\right)}$, $a_{\beta ,1}\left(l,i\right)$, and $a_{\beta ,2}\left(l,i\right)$ are all constant.

When the target does not have a parent node, its motion is not affected by other targets, and we call it a head node. The displacement vector $b_{k}\left(l,i\right)=0$ if the target does not have a parent node. If the target has multiple parent nodes and obeys linear motion, the model is expressed as:

(23) $$\begin{array}{ccc} \;\;x_{k+1,i}\;\; & = & \;\;\;\sum_{l\in P(i)}\omega_{k}\left(l,i\right)\left[F_{k,l}x_{k,l}\;+\;b_{k}\left(l,i\right)\right]\;+\Gamma_{k,i}\omega_{k,i} \end{array}$$

(24) $$\begin{array}{ccc} x_{k,i} & \in & X_{k},\sum_{l\in P(i)}\omega_{k}\left(l,i\right)=1,\omega_{k,i}\in \left[0,1\right]. \end{array}$$

According to Reference [52], when all targets have the same transition matrix and some others condition hold, $F_{k,l}=F_{k}$. This is available from Equation (10):

(25) $$x_{k+1,i}=F_{k}x_{k,i}+\Delta b_{k}\left(l,i\right)+\Gamma_{k,i}\omega_{k,i},$$

where $\Delta b_{k}\left(l,i\right)=\sum_{l\in P(i)}\omega_{k}\left(l,i\right)\left[b_{k}\left(l,i\right)-F_{k}{\overset{ˇ}{b}}_{k}\left(l,i\right)\right]$, ${\overset{ˇ}{b}}_{k}\left(l,i\right)=x_{k,i}-x_{k,l}$.

In Reference [52], a new collaborative noise is proposed:

(26) $$\begin{array}{c} \omega_{k,i}^{o}=\Delta b_{k}\left(l,i\right)+\Gamma_{k,i}\omega_{k,i} \end{array}.$$

Equation (26) suggests that the new noise is only influenced by the collaborative noise. So for each target *i*, we can build new model from the target state $x_{k,i}$, adjacency matrix $A_{d}$, and collaborative noise $\omega_{k,i}^{o}$, using the following a proposition. Before this, a definition is first introduced.

**Definition** **1** (Reference [52])**.** *A movement of group is said to be simple if it meets the following conditions:*

- The movement equations are all linear and same, i.e., $F_{k,l}=F_{k}$.
- The movement mode is CV, CT with known turning rate, or the constant acceleration (CA).
- The formation of group targets are fixed and, thus, the displacement vector only exists in the position displacement, i.e., $b_{k}\left(l,i\right)=\left[b_{k,x}\left(l,i\right),0,b_{k,y}\left(l,i\right),0\right]$.
- The collaboration relation of individual targets are of tree graph. This means each vertex has only one father vertex.

**Proposition** **1** (Reference [52])**.** *Suppose that the dynamic model of group targets is given by Equations *(11)*, *(23)*, *(24)*. If the following conditions hold: (1) The group targets’ movement are simple; (2) the displacement vector $\left\{b_{k}\left(l,i\right)\right\}$ is Gaussian, i.e.,*

(27) $$\begin{array}{ccc} \;\;\omega_{k,i}^{o}\;\; & ∼ & \;\;N(0,Q_{k}^{0}\left(l,i\right)) \end{array}$$

(28) $$\begin{array}{ccc} \;\;Q_{k,i}^{0}\;\; & = & \;\;F_{k}\left[P_{k,l}-P_{k,i}\right]F_{k}^{T}+S_{k}\left(l,i\right)+\Gamma_{k,i}Q_{k,i}\Gamma_{k,i}^{T}. \end{array}$$

*It follows from Equation *(27)* that the collaborative noise $\omega_{k,i}^{o}$ is Gaussian with zero-mean and covariance $Q_{k,i}^{0}$. It follows from Equation *(28)* that the acquisition of $Q_{k,i}^{0}$ depends on the adjacency matrix $A_{d}$, so we can write the state transition probability in the following form:*

(29) $$f\left(x_{k+1,i}\left|x_{k,i},A_{k}\right.\right)=N\left(x_{k+1,i},F_{k,i}x_{k,i},Q_{k,i}^{o}\right).$$

Based on Proposition 1, when a group moves in a maneuvering model, a further result can be given as follows:

**Proposition** **2.** *Suppose that the dynamic model of group targets is given by Equations *(11)*, *(23)*, *(24)*. If the group targets’ movement are simple and target i with a parent l then:*

(30) $$\begin{array}{ccc} \;\;\omega_{k,i}^{o}\;\; & ∼ & \;\;N(\mu_{k}^{0}\left(l,i\right),Q_{k}^{0}\left(l,i\right)) \end{array}$$

(31) $$\begin{array}{ccc} \;\;\mu_{k}^{0}\left(l,i\right) & = & \;\;\left[c_{k}\left(l,i\right)C_{a}\left(l,i\right){\hat{x}}_{k,l}-F_{k}\left({\hat{x}}_{k,i}-{\hat{x}}_{k,l}\right)\right] \end{array}$$

(32) $$\begin{array}{ccc} \;\;Q_{k,i}^{0}\;\; & = & \;\;F_{k}\left[P_{k,l}-P_{k,i}\right]F_{k}^{T}+c_{k}^{2}\left(l,i\right)C_{a}\left(l,i\right)P_{k,l}C_{a}^{T}\left(l,i\right)+\Gamma_{k,i}Q_{k,i}\Gamma_{k,i}^{T} \end{array}$$

*It follows from Equations *(30)*, *(31)*, *(32)* that the collaborative noise $\omega_{k,i}^{o}$ is Gaussian with $\mu_{k}^{0}\left(l,i\right)$ and covariance $Q_{k,i}^{0}$.*

**Remark** **1.** *It should be noted that the mean $\mu_{k}^{0}\left(l,i\right)$ is the bias of the designed displacement $b_{k}\left(l,i\right)$ and estimated displacement $F_{k}\left({\hat{x}}_{k,i}-{\hat{x}}_{k,l}\right)$. For a fixed formation, i.e., the matrix $C_{a}\left(l,i\right)$ is constant, the coefficient $c_{k}\left(l,i\right)$ is commonly time-varying for maneuver movement. However, if parent node velocity $\sqrt{{\dot{p}}_{k,x}^{2}\left(l\right)+{\dot{p}}_{k,y}^{2}\left(l\right)}$ can be gotten, then the coefficient $c_{k}\left(l,i\right)$ can be estimated. For example, for CT movement, the parent node velocity is a constant. Otherwise, the predicited value can be adopted.*

Another point is the relation between covariance and adjacency matrix. From Equations (31), (32), to calculate the means and covariance, the parent vertex *l* should be first known. This is dependent on adjacency matrix Equation (6). In this paper, the adjacency matrix is defined on the label space and known in prior. That is, in the predicted stage, the adjacency matrix can be gotten and adopted according to the predicted labels. In contrast, if the adacency matrix is unknown, and it needs to be estimated according to the predicted states. In general, the adacency relation is based on the target states, or the motion information. A detailed discussion can be found in Reference [52].

#### 3.2.1. State Prediction

For a resolvable group target $x_{k}$, its prediction density is:

(33) $$p\left(x_{k+1,i}\right)=\int N\left(x_{k+1,i},F_{k,i}x_{k,i},Q_{k,i}^{o}\right)p\left(x_{k,i}\right)dx_{k,i}.$$

If $p\left(x_{k,i}\right)=N\left(x_{k,i},\mu_{k,i},P_{k,i}\right)$, then:

(34) $$\begin{array}{ccc} p(x_{k+1,i}) & = & N(x_{k+1,i},\mu_{k+1\left|k\right.,i},P_{k+1\left|k\right.,i}) \end{array}$$

(35) $$\begin{array}{ccc} \mu_{k+1\left|k\right.,i} & = & F_{k,i}x_{k,i} \end{array}$$

(36) $$\begin{array}{ccc} P_{k+1\left|k\right.,i} & = & F_{k,i}P_{k,i}F_{k,i}^{T}+Q_{k,i}^{o}. \end{array}$$

Therefore, the state covariance is related to the adjacency matrix $A_{d}$.

#### 3.2.2. State Update

The predicted density $p_{k+1,i}$ is Gaussian, and the corresponding posterior function is:

(37) $$p\left(x_{k+1,i}\left|z_{k+1}\right.\right)=\frac{g\left(z_{k+1}\left|x_{k+1}\right.\right)p\left(x_{k+1,i}\right)}{\int g\left(z_{k+1}\left|x_{k+1}\right.\right)p\left(x_{k+1,i}\right)dx_{k+1,i}}.$$

The numerator of Equation (37) is derived by:

(38) $$\begin{array}{ccc} & & g\left(z_{k+1}\left|x_{k+1}\right.\right)p\left(x_{k+1,i}\right)\\ \; & = & \;\;N\left(z_{k\;+\;1},H_{k\;+\;1}x_{k\;+\;1},R_{k\;+\;1}\right)N\left(x_{k\;+\;1,i},\mu_{k\;+\;1\left|k\right.,i},P_{k\;+\;1\left|k\right.,i}\right)\\ \; & = & \;\;q\left(z_{k+1}\right)N\left(x_{k+1,i},\mu_{k+1,i},P_{k+1,i}\right), \end{array}$$

where

(39) $$\begin{array}{ccc} q(z_{k+1})\;\;\; & = & \;\;\;N\left(z_{k\;+\;1},H_{k\;+\;1}\mu_{k\;+\;1\left|k,i\right.},R_{k\;+\;1}\;+\;H_{k\;+\;1\;}P_{k\;+\;1,i}\right)H_{k\;+\;1,i}^{T} \end{array}$$

(40) $$\begin{array}{ccc} \mu_{k+1,i}\;\;\; & = & \;\;\;\mu_{k+1\left|k,i\right.}+K_{k+1,i}\left(z_{k+1}-H_{k+1}\mu_{k+1\left|k,i\right.}\right) \end{array}$$

(41) $$\begin{array}{ccc} P_{k+1,i}\;\;\; & = & \;\;\;\left(I-K_{k+1,i}H_{k+1}\right)P_{k+1\left|k\right.,i} \end{array}$$

(42) $$\begin{array}{ccc} K_{k+1,i}\;\;\; & = & \;\;\;P_{k+1,i}H_{k+1}{\left(H_{k+1}P_{k+1,i}H_{k+1}^{T}+R_{k+1}\right)}^{-1}. \end{array}$$

## 4. The GLMB Filter for Resolvable Group Targets

### 4.1. The GLMB Filter

Under the standard multi-target transition and measurement model, the δ-GLMB filter [14,15] is an exact solution to the optimal Bayes multi-target filter. First, let:

(43) $$\begin{array}{ccc} \;\;C & = & F\left(L\right)\times \Xi \end{array}$$

(44) $$\begin{array}{ccc} \omega^{\left(c\right)}\left(L\right) & = & \omega^{\left(I,\xi \right)}\left(L\right)=\omega^{\left(I,\xi \right)}\delta_{I}\left(L\right) \end{array}$$

(45) $$\begin{array}{ccc} p^{\left(c\right)} & = & p^{\left(I,\xi \right)}=p^{\xi }, \end{array}$$

and let π be a δ-GLMB density:

(46) $$\pi \left(X\right)=\Delta \left(X\right)\sum_{\left(I,\xi \right)\in F\left(L\right)\times \Xi }\omega^{\left(I,\xi \right)}\delta_{I}\left(L\left(X\right)\right){\left[p^{\left(\xi \right)}\right]}^{X}.$$

The δ-GLMB prediction density to time k+1 is given by:

(47) $$\pi_{+}\left(X_{+\;}\right)=\Delta \left(X_{+\;}\right)\;\;\;\sum_{\left(I,\xi \right)\in F\left(L\right)\times \Xi }\;\;\;\;{\omega_{+}}^{\left(I,\xi \right)}\delta_{I_{+}}\left(L\left(X_{+}\right)\right)\;{\left[{p_{+}}^{\;\left(\xi \right)}\right]}^{X_{+}},$$

where

(48) $$\begin{array}{ccc} \;\;\omega_{+}^{\left(I_{+},\xi \right)}\;\; & = & \;\;\omega_{B}\left(I_{+}\cap B\right)\omega_{S}^{\xi }\left(I_{+}\cap L\right) \end{array}$$

(49) $$\begin{array}{ccc} \;\;p_{S}^{\left(\xi \right)}\left(x,l\right)\;\; & = & \;\;1_{L}\left(l\right)p_{s}^{\left(\xi \right)}\left(x,l\right)+\left(1-1_{L}\left(l\right)\right)p_{B}\left(x,l\right) \end{array}$$

(50) $$\begin{array}{ccc} \;\;p_{s}^{\left(\xi \right)}\left(x,l\right)\;\; & = & \;\;\frac{\langle p_{S}\left(\cdot ,l\right)f\left(x\left|\cdot \right.,l\right),p^{\left(\xi \right)}\left(\cdot ,l\right)\rangle }{\eta_{S}^{\left(\xi \right)}\left(l\right)} \end{array}$$

(51) $$\begin{array}{ccc} \;\;\eta_{S}^{\left(\xi \right)}\left(l\right)\;\; & = & \;\;\int \langle p_{S}\left(\cdot ,l\right)f\left(x\left|\cdot ,l\right.\right),p^{\left(\xi \right)}\left(\cdot ,l\right)\rangle dx \end{array}$$

(52) $$\begin{array}{ccc} \;\;\omega_{s}^{\left(\xi \right)}\left(L\right)\;\; & = & \;\;{\left[\eta_{S}^{\left(\xi \right)}\right]}^{L}\sum_{I\subseteq L}1_{I}\left(L\right){\left[q_{S}^{\left(\xi \right)}\right]}^{I-L}\omega^{\left(I,\xi \right)} \end{array}$$

(53) $$\begin{array}{ccc} \;\;q_{S}^{\left(\xi \right)}\left(l\right)\;\; & = & \;\;\langle q_{S}\left(\cdot \left|l\right.\right),p^{\left(\xi \right)}\left(\cdot ,l\right)\rangle . \end{array}$$

Note that $\omega_{B}\left(I_{+}\cap B\right)$ is the weight of the birth labels $\left(I_{+}\cap B\right)$, and $\omega_{S}^{\xi }\left(I_{+}\cap L\right)$ is the weight of the survival labels $\left(I_{+}\cap L\right)$. $p_{B}\left(\cdot ,l\right)$ is the density of the newly born target, and $p_{s}^{\left(\xi \right)}\left(x,l\right)$ is the predicted density of a surviving target.

The update step is the same as the original formulation:

(54) $$\pi \left(X\left|Z\right.\right)\approx \Delta \left(X\right)\sum_{\left(I,\xi \right)\in F\left(L\right)\times \Xi }\sum_{\theta \in \Theta^{\left(M\right)}}{\tilde{\omega }}^{\left(I,\xi ,\theta \right)}\delta_{I}\left(L\left(X\right)\right){\left[p^{\left(\xi ,\theta \right)}\right]}^{X},$$

for each $\left(I,\;\;\;\xi \;\;\;\right)$,${\;\;\;\Theta \;\;\;}^{\left(M\right)}=\left\{\zeta^{\left(1\right)},\cdots ,\zeta^{\left(M\right)}\right\}$ is the element *M* of the highest weight Θ. ${\tilde{\omega }}^{\left(I,\xi ,\theta^{\left(i\right)}\right)}$, $\omega^{\left(I,\xi ,\theta^{\left(i\right)}\right)}$ is the weight after truncation.

### 4.2. The UKF GLMB Filter

The constant turn model is a nonlinear motion model with unknown turning rate. The unscented Kalman filter (UKF) is an algorithm for nonlinear filtering proposed in Reference [57]. This paper used the UKF to perform prediction and update for individual track following the CT model in the GLMB filter algorithm. The UKF filtering algorithm is summarized in Table 1.

### 4.3. Efficient Implementation of the GLMB Filter

The Gibbs GLMB algorithm [15] combines the prediction and update steps of the GLMB filter, which effectively improves the efficiency of the truncation process. So, we introduced it to solve the problem of resolvable group target tracking under nonlinear condition.

(55) $$\pi_{k}\left(X\right)\propto \Delta \left(X\right)\sum_{J,\xi ,\theta }\left(\sum_{I}\omega_{k-1}^{\left(I,\xi \right)}\omega_{k}^{\left(I,\xi ,J,\theta \right)}\left(Z_{k}\right)\right)\delta_{J}\left(L\left(X\right)\right){\left[{p_{k}}^{\left(\xi ,\theta \right)}\left(\cdot |Z_{k}\right)\right]}^{X},$$

where

$$\omega_{k}^{\left(I,\xi ,J,\theta \right)}\left(Z_{k}\right)=1_{\Theta_{k}\left(J\right)}\left(\theta \right){\left[1-r_{B,k}\right]}^{B_{k}-J}{\left[r_{B,k}\right]}^{B_{k}\cap J}{\left[1-{\bar{P}}_{S,k}^{\left(\xi \right)}\right]}^{I-J}{\left[{\bar{P}}_{S,k}^{\left(\xi \right)}\right]}^{I\cap J}{\left[{\bar{\Psi }}_{Z_{k},k}^{\left(\xi ,\theta \right)}\right]}^{J}$$

$${\bar{\Psi }}_{Z_{k},k}^{\left(\xi ,\theta \right)}\left(l\right)=\langle \Psi_{Z_{k},k}^{\left(\xi ,\theta (l)\right)}\left(\cdot ,l\right),p_{k|k-1}^{\left(\xi \right)}\left(\cdot ,l\right)\rangle$$

$${\bar{P}}_{S,k}^{\left(\xi \right)}\left(l\right)=\langle P_{S,k}\left(\cdot ,l\right),p_{k-1}^{\left(\xi \right)}\left(\cdot ,l\right)\rangle$$

$${p_{k}}^{\left(\xi ,\theta \right)}\left(x,\cdot |Z_{k}\right)\propto \Psi_{Z_{k},k}^{\left(\xi ,\theta (l)\right)}\left(x,l\right)p_{k|k-1}^{\left(\xi \right)}\left(x,l\right)$$

$$p_{k|k-1}^{\left(\xi \right)}\left(x,l\right)=1_{B_{k}}\left(l\right)p_{(}B,k)\left(x,l\right)+1_{L_{k-1}}\left(l\right)\frac{\langle P_{S,k}\left(\cdot ,l\right)f_{k|k-1}\left(x|\cdot ,l\right),p_{k-1}^{\left(\xi \right)}\left(\cdot ,l\right)\rangle }{{\bar{P}}_{S,k}^{\left(\xi \right)}\left(l\right)},$$

Truncation by sampling ${\left\{\left(I^{\left(i\right)},\xi^{\left(i\right)},J^{\left(i\right)},\theta^{\left(i\right)}\right)\right\}}_{i=1}^{H_{k,max}}$ from some distribution π. It should be noted in the predicted density $p_{k|k-1}^{\left(\xi \right)}\left(\cdot ,l\right)$ that the collaboration noise $\omega_{k,i}^{o}$ (Equation (30)) is adopted, instead of process noise $\omega_{k,i}$, as in Equation (25).

### 4.4. The Algorithm Implementation and Settings

The group target state estimation is complicated due to the limited observations and collaboration between targets. In this paper, we defined the collaboration on the labels of individual targets. Although a target label is just a “temporary identity”, the identity contains the collaboration information (group structure) modeled in the adjacency matrix. We tried to use the “temporary identity” to get the group structure and show it is important in estimating target states if it is known in prior.

Therefore, the adjacency matrix was assumed to be known. In contrast, the number of targets and sub-groups were all unknown and needed to be estimated. For simplicity, we did not consider the estimation of sub-groups, which was considered in Reference [52]. Interested readers can refer to it for further information.

## 5. Simulations

For this simulation experiment, the radar sensor is adopted to track group targets. The UKF-GLMB and UKF-Gibbs GLMB filters are used for comparison. Two experiments were compared in this section. In experiment 1, two filters were used to track group targets with cooperative. And in experiment 2, they were used to track group targets with cooperative to non-cooperative. In both simulations, the state vector is $x_{k}=\left[p_{k,x},{\dot{p}}_{k,x},p_{k,y},{\dot{p}}_{k,y}\right]$. The dynamic function and the radar observations are given by:

(56) $$\begin{array}{c} x_{k+1,i}=F_{k,l}\left(\omega_{k}\right)x_{k,l}+b_{k}\left(l,i\right)+\Gamma_{k,i}w_{k,i} \end{array}$$

(57) $$\begin{array}{c} z_{k+1}=\left[\begin{array}{c} r_{k+1}\\ \theta_{k+1} \end{array}\right]=\left[\begin{array}{c} \sqrt{p_{k+1,x}^{2}+p_{k+1,y}^{2}}+v_{k+1,r}\\ arctan\frac{p_{k+1,y}}{p_{k+1,x}}+v_{k+1,\theta } \end{array}\right], \end{array}$$

where:

(58) $$F_{k,l}\left(\omega_{k}\right)=\left[\begin{array}{cccc} 1 & \sin\omega_{k}/\omega_{k} & 0 & -\left(1-\cos\omega_{k}\right)/\omega_{k}\\ 0 & \cos\omega_{k} & 0 & -\sin\omega_{k}\\ 0 & \left(1-\cos\omega_{k}\right)/\omega_{k} & 1 & \sin\omega_{k}/\omega_{k}\\ 0 & -\sin\omega_{k} & 0 & \cos\omega_{k} \end{array}\right].$$

And the initial state of the two parent targets are: $x_{1,1}=\left[-1500;30;200;25\right],x_{2,1}=\left[1500;-30;750;30\right]$.

### 5.1. Scenario 1

**Figure 4:**
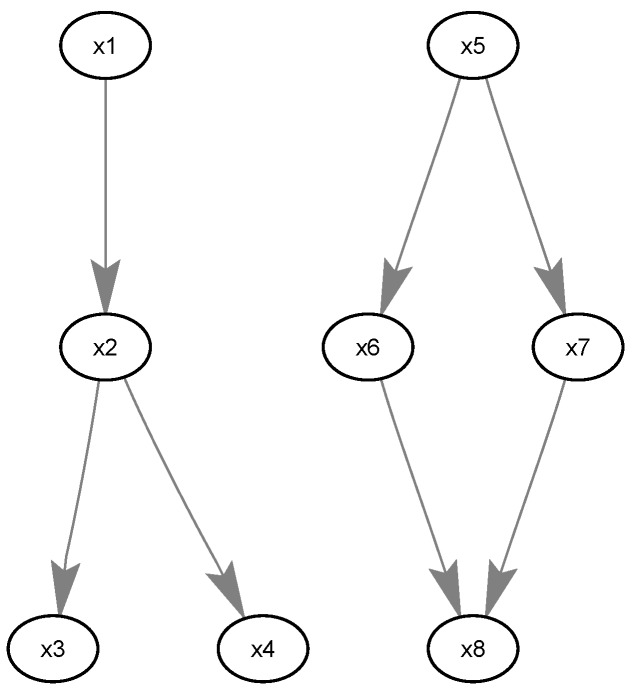
The structures of two sub-groups.

For this simulation, we used Gibbs GLMB filter and GLMB filter for comparison. In the simulation, the group targets are shown in Figure 4, including two sub-groups.

The distance between any parent and its child vertices was 100 m. Each sub-group target contained four targets, i.e., $\left\{\left(x_{1},\ell_{1}\right),\cdots ,\left(x_{4},\ell_{4}\right)\right\}$ as sub-group 1 and $\left\{\left(x_{5},\ell_{5}\right),\cdots ,\left(x_{8},\ell_{8}\right)\right\}$ as sub-group 2. The two sub-groups are independent of each other. Let the adjacency matrices for the two sub-groups be known and given by:

(59) $$A(\ell_{1},\cdots ,\ell_{4})=\left[\begin{array}{cccc} 0 & 0 & 0 & 0\\ 1 & 0 & 0 & 0\\ 0 & 1 & 0 & 0\\ 0 & 1 & 0 & 0 \end{array}\right],A(\ell_{5},\cdots ,\ell_{8})=\left[\begin{array}{cccc} 0 & 0 & 0 & 0\\ 1 & 0 & 0 & 0\\ 1 & 0 & 0 & 0\\ 0 & 1 & 1 & 0 \end{array}\right].$$

**Figure 5:**
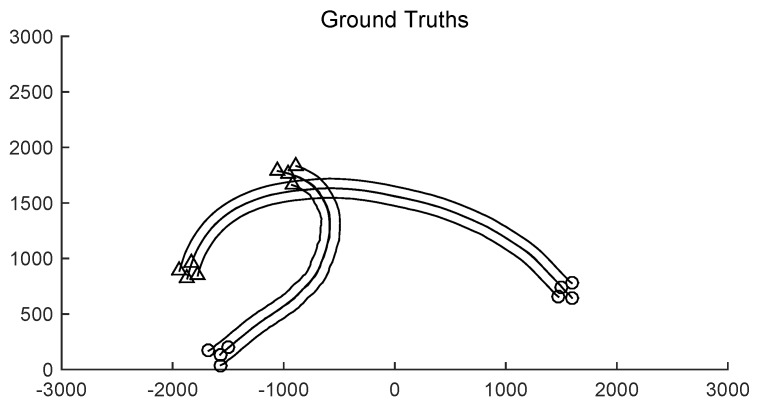
The tracks of group targets.

The monitoring range of the experiment was [−π/2,π/2; 0 m, 3000 m]. The experiment lasted 100 s, sub-group 1 was born at the time of *k* = 0 s and disappeared at the time of *k* = 70 s, and sub-group 2 was born at k=20 s and disappeared at *k* = 100 s. The covariance of the observed noise R=diag[0.0012100]. The covariance of process noise Q=diag[0.040.040.04]. The real trajectory of the target is shown in Figure 5. The curve represents the trajectory, the circle represents the starting point, and the triangle represents the end point. In this experiment, GLMB and Gibbs GLMB were used to estimate them, respectively.

**Figure 6:**
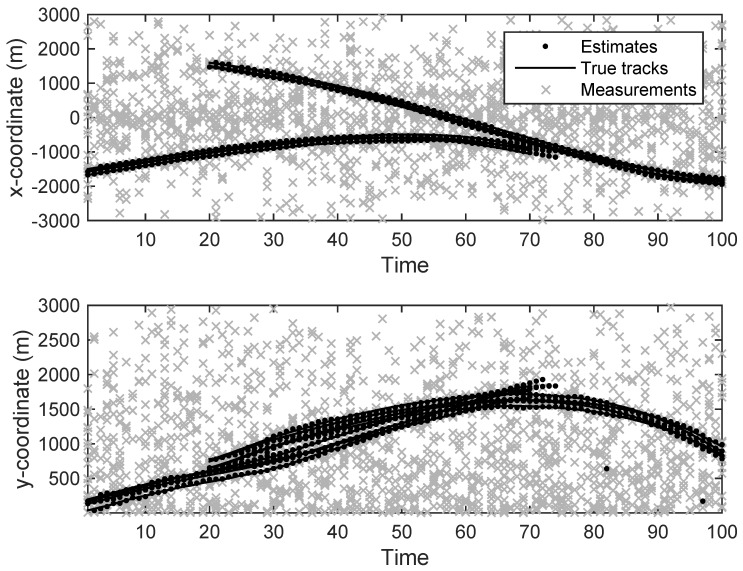
The state estimation by Generalized Labeled Multi-Bernoulli (GLMB) filter.

**Figure 7:**
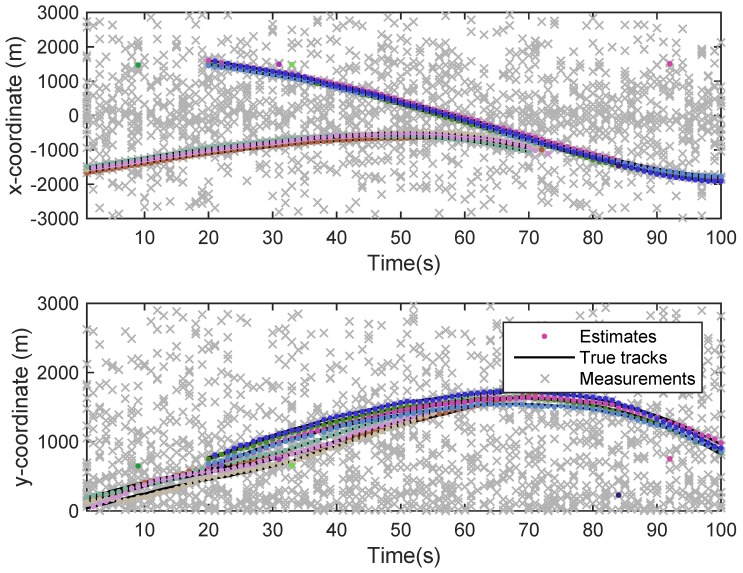
The state estimation by Gibbs GLMB filter.

**Figure 8:**
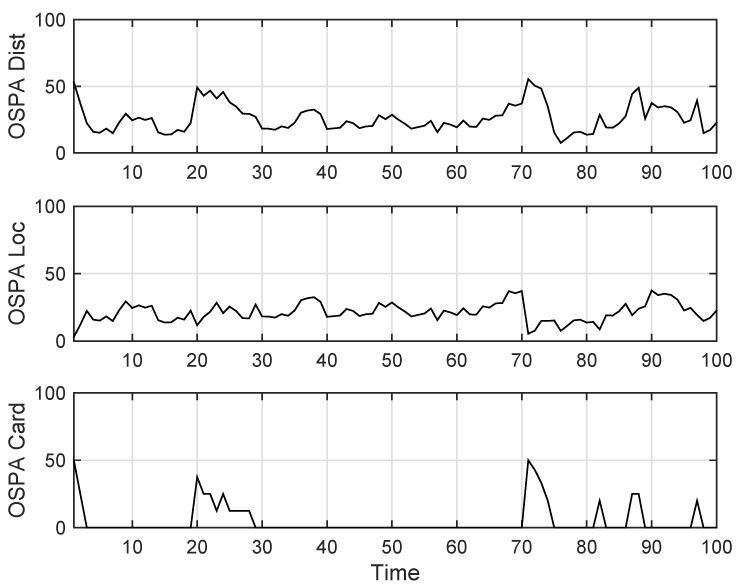
The OSPA distance by GLMB filter.

**Figure 9:**
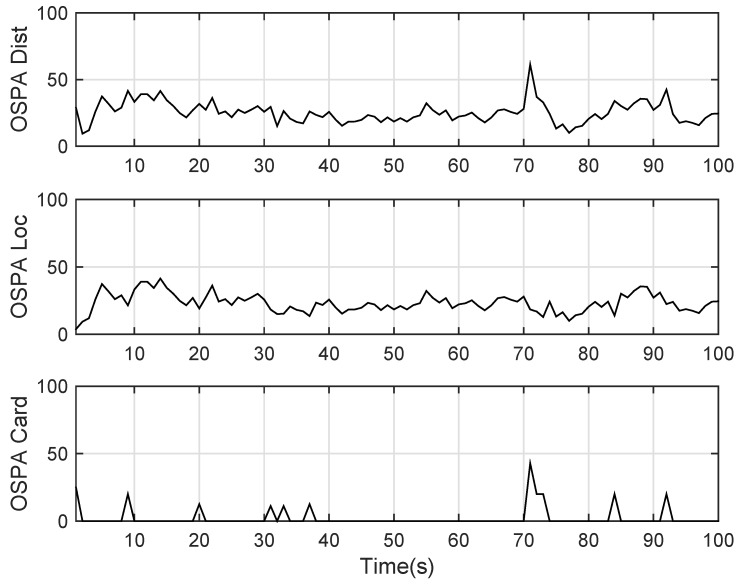
The OSPA distance by Gibbs GLMB filter.

**Figure 10:**
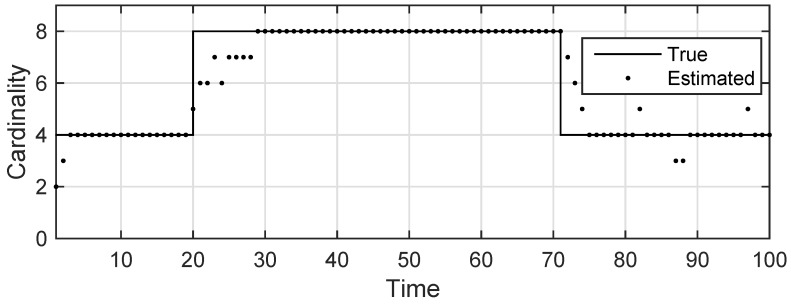
The estimated number of targets by GLMB filter.

**Figure 11:**
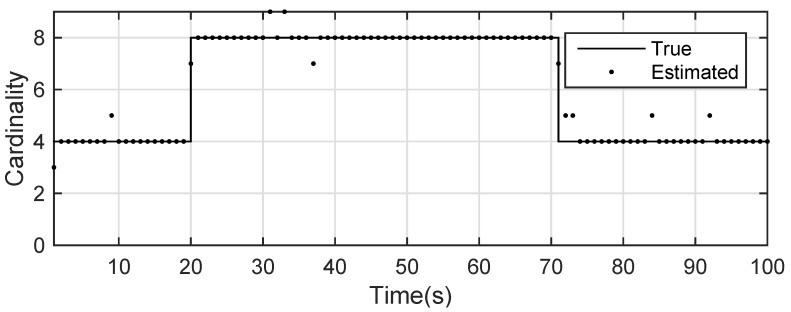
The estimated number of targets by Gibbs GLMB filter.

The state estimation obtained by UKF-GLMB filtering algorithm is shown in Figure 6 and by UKF-Gibbs GLMB filtering is shown in Figure 7, the OSPA distance is shown in Figure 8 and by UKF-Gibbs GLMB filtering is shown in Figure 9. The number of targets is estimated in Figure 10 and by UKF-Gibbs GLMB filtering is shown in Figure 11.

It can be seen from Figure 6 and Figure 7 that both filters could accurately estimate the motion state of each target at each moment. It can be seen from the OSPA loc and OSPA Dist in Figure 8 and Figure 9 and Table 3 that the Gibbs GLMB filter was better in the state estimation effect of the target. It can be seen from the OSPA Loc and OSPA Dist in Figure 8 and Figure 9 that the Gibbs GLMB filter was better in the state estimation effect of the target. For the estimation of the number of targets, it can be seen from Figure 10 and Figure 11 that both could effectively track the number of targets, but Gibbs GLMB performed better, and it can be seen from the OSPA Cad in Figure 8 and Figure 9 that the delay time of SCA-GLMB in the tracking process was less than the GLMB filter. In addition, we recorded the running time of the two filtering algorithms in ten experiments, as shown in Table 3. As can be seen from Table 1, the time required for the GLMB filtering algorithm to run once was 264.5118 s, while that for Gibbs GLMB was 43.4962 s. So, we can see that the average efficiency of the Gibbs GLMB filter was more than six times faster than the GLMB filter.

### 5.2. Scenario 2

**Figure 12:**
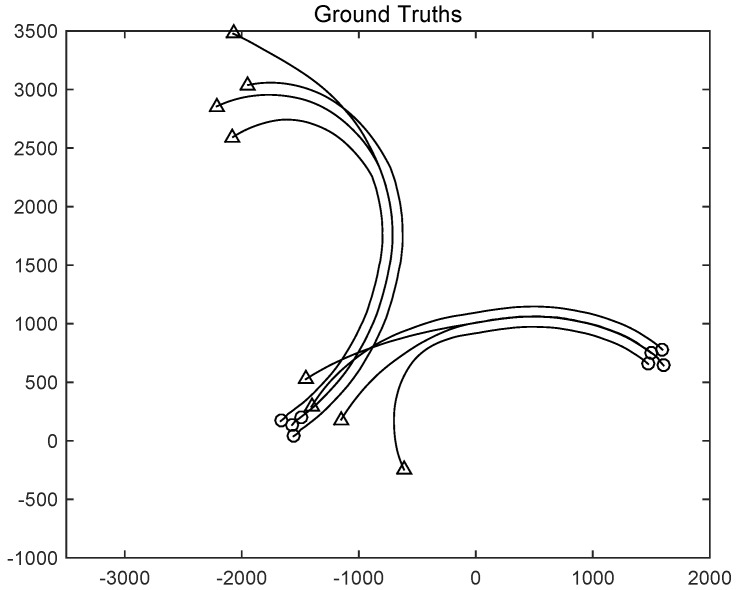
The tracks of group targets for experiment 2.

In this subsection, the group targets, including two sub-groups, are shown in Figure 12. Similarly, each sub-group target contained four sub-targets and the adjacency matrix was the same as Scenario 1. The two sub-groups were mutually independent. Sub-group 1 was born at time *k* = 1 s and survived in [1 s, 100 s]. Sub-group 2 was born at *k* = 10 s and survived in time interval [10 s, 100 s]. The sub-group collaborate in time interval [1 s, 60 s] and without the collaboration after 60 s step, i.e., [61 s, 100 s]. The covariance of the observed noise R=diag[0.0012100]. The covariance of process noise Q=diag[0.040.040.04]. The real trajectory of the target is shown in Figure 12, where the curve shows the trajectory, the circle represents the starting point, and the triangle is the end points.

**Figure 13:**
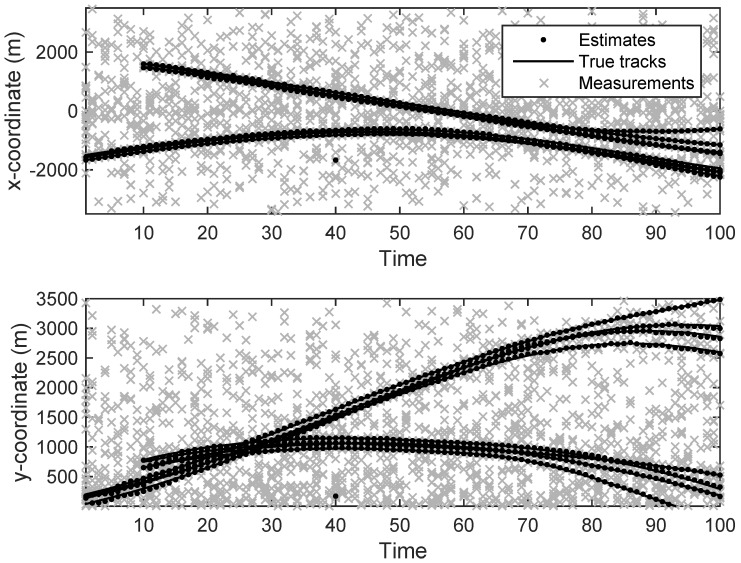
The state estimation by GLMB filter for experiment 2.

**Figure 14:**
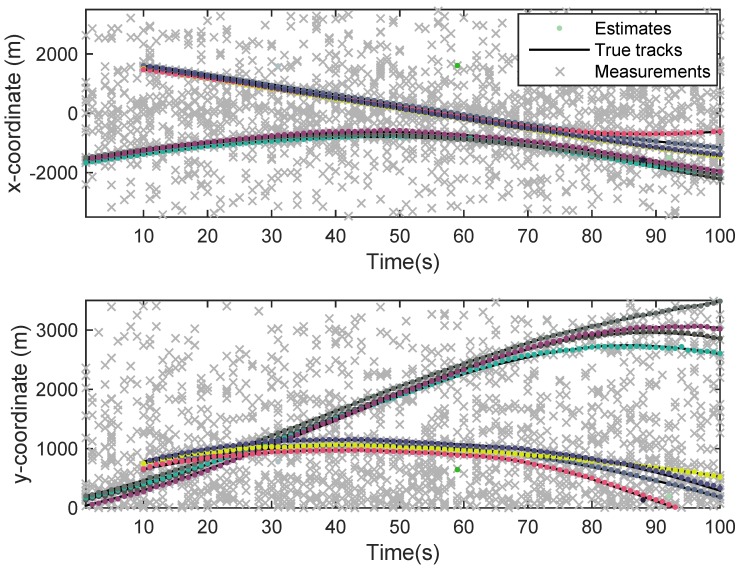
The state estimation by Gibbs GLMB filter for experiment 2.

**Figure 15:**
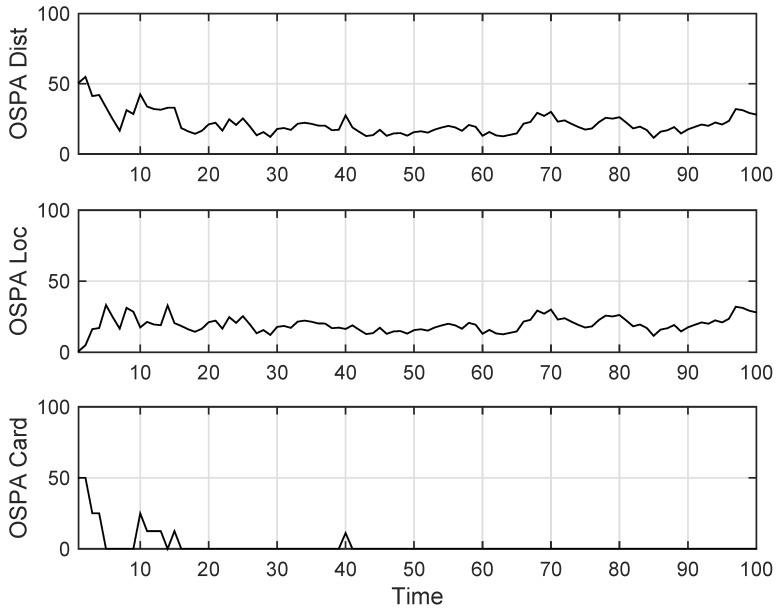
The OSPA distance by GLMB filter for experiment 2.

**Figure 16:**
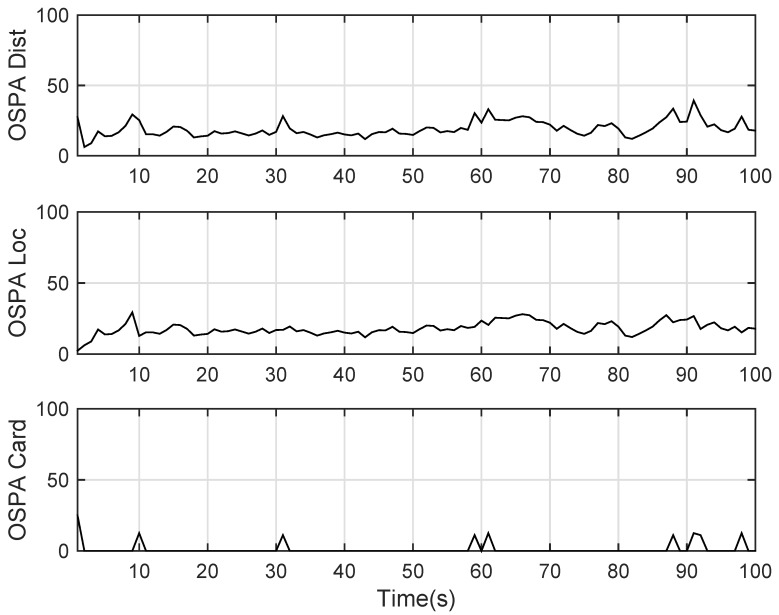
The OSPA distance by Gibbs GLMB filter for experiment 2.

**Figure 17:**
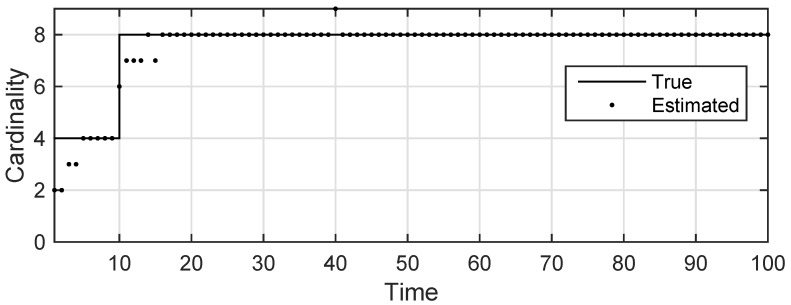
The estimated number of targets by GLMB filter for experiment 2.

**Figure 18:**
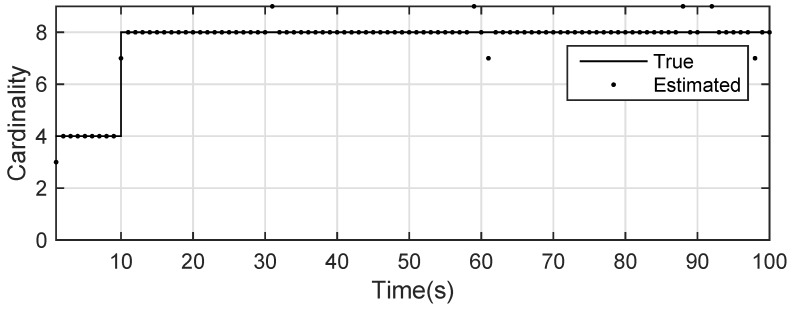
The estimated number of targets by Gibbs GLMB filter for experiment 2.

The state estimation obtained by UKF-GLMB filtering is shown in Figure 13 and the UKF Gibbs GLMB filtering is shown in Figure 14. The OSPA distance is shown in Figure 15 and by UKF Gibbs GLMB filtering is shown in Figure 16. The number of targets is shown in Figure 17 and the UKF Gibbs GLMB filtering is shown in Figure 18.

From Figure 13 and Figure 14, we know that both filters can accurately estimate the motion state of each target. The Gibbs GLMB filter was better in the state estimation as shown by Figure 15 and Figure 16. For the number of targets, it can be seen from Figure 17 and Figure 18 that both can effectively estimate the number of targets and Gibbs GLMB performed better in speed. This can be seen from the OSPA metric plotted in Figure 15 and Figure 16. As in Scenario 1, we recorded the running time of the two algorithms. In ten experiments, as shown in Table 4, the time cost for GLMB filtering was 284.1470 s, while for Gibbs GLMB it wa 23.7307 s. In tracking performance, the two algorithms had a close performance.

## 6. Conclusions

In this paper, we concentrated on multiple resolvable group targets tracking. Different from the previous work, we considered the collaboration relation of the group. We incorporated graph theory into labeled RFS to model collaborative dependence between targets in the same group. Based on original GLMB filter and Gibbs GLMB filter, we considered the resolvable group target estimation, where the new collaboration noise under maneuver was modeled. Simulations on nonlinear example validatde the soundness of the proposed algorithm.

## Figures and Tables

**Table 1:** The UKF GLMB Algorithm [19].

1. Given the initial sigma point, as shown below:
${\left\{\omega_{0,n}^{\pm (j,i)},s_{0,n}^{\pm (j,i)}\right\}}_{i=1}^{J_{\Gamma ,k}^{(j),n}},\Phi_{k}\left(\cdot \right),h_{k}\left(\cdot \right)$.
2. Sigma parameter point prediction:
(1) Matrix parameter $s_{k\left|k-1\right.,n}^{\left(j\right)}$, $P_{k\left|k-1\right.,n}^{j}$ and target existence probability $r_{k\left|k-1\right.,n}$ can be seen in Table 2.
(2) Other parameters $S_{k\left|k-1\right.,n}^{\left(j\right)}$, $C_{k,n}^{\left(j\right)xz}$ and $K_{k,n}^{\left(j\right)}$ can be seen in Table 2.
(3) Sigma point parameter update.
3. Get target status ${}^{o}$${\hat{x}}_{k,n}^{\left(j\right)}$; covariance matrix $P_{k,n}^{\left(j\right)}$; target existence probability $r_{k,n}^{\left(j\right)}$ seen in Table 2.

**Table 2:** UKF Algorithm Step [19].

(1) Initial sigma point ${}^{o}$
$s_{k-1,n}^{0}={\left[x_{k-1,n}^{T},\omega_{k-1,n}^{T}\right]}^{T}$
$s_{k-1,n}^{\pm i}=a_{k-1,n}^{0}\pm {\left(\sqrt{\left(n_{s}+\beta \right)diag\left(P_{k-1,n},Q_{k-1,n}\right)}\right)}_{i}$
$\omega_{k-1}^{0}=\beta /\left(n_{s}+\beta \right)$,$\omega_{k-1}^{\pm i}=1/2\left(n_{s}+\beta \right),i=1,\cdots ,n_{s}$
(2) Predictive step
$s_{k,n}^{i}=\Phi_{k}\left(s_{k-1,n}^{i}\right)+\omega_{k-1,n},s_{k\left|k-1\right.,n}=\sum \omega_{k-1,n}^{i}s_{k,n}^{m}$
$P_{k\left|k-1\right.,n}=\sum \omega_{k-1,n}^{i}\left(s_{k,n}^{i}-s_{k\left|k-1\right.,n}\right)\left(s_{k,n}^{i}-s_{k\left|k-1\right.,n}\right)$
$b_{k,n}^{0}={\left[s_{k\left|k-1,n\right.}^{T},{\bar{v}}_{k}^{T}\right]}^{T}$
$b_{k-1,n}^{\pm }=b_{k-1,n}^{0}\pm {\left(\sqrt{\left(\beta \right)diag\left(P_{k\left|k-1\right.,n},R_{k}\right)}\right)}_{i}$
$z_{k,n}^{i}=h_{k}\left(b_{k,n}^{i}\right),z_{kk-1,n}=\sum \omega_{k,n}^{i}z_{k,n}^{i}$
$S_{k,n}=\sum \omega_{k-1,n}^{i}\left(z_{k,n}^{i}-z_{k\left|k-1\right.,n}\right){\left(z_{k,n}^{i}-z_{k\left|k-1\right.,n}\right)}^{T}$
$C_{k,n}=\sum \omega_{k-1,n}^{i}\left(s_{k,n}^{m}-s_{k\left|k-1,n\right.}\right){\left(z_{k,n}^{i}-z_{k\left|k-1\right.,n}\right)}^{T}$
$K_{k,n}=C_{k,n}^{xz}S_{k,n}^{-1}$
(3) Update step
${\hat{x}}_{k,n}=s_{k\left|k-1,n\right.}+K_{k}\left(z_{k,n}-z_{k\left|k-1\right.}\right)$
$P_{k,n}=P_{k\left|k-1\right.,n}-K_{k,n}S_{k,n}K_{k,n}^{T}$

**Table 3:** The running time and precision of filters (the average of 10 times).

	Time (s)		Precision (m)	
	GLMB	Gibbs GLMB	GLMB	Gibbs GLMB
Data	264.5118	43.4962	28.8332	28.0190

**Table 4:** The running time and precision of filters (the average of 10 times) for experiment 2.

	Time (s)		Precision (m)	
	GLMB	Gibbs GLMB	GLMB	Gibbs GLMB
Data	284.1740	39.1331	23.7307	19.4597
